# Prognostic value of endothelial activation and stress index in mechanical thrombectomy for patients with acute ischemic stroke

**DOI:** 10.3389/fnagi.2025.1683690

**Published:** 2025-12-10

**Authors:** Xiaohua Zhu, Yunnan Lu, Yaojia Xu, Yongxin Li, Qingyong Dai, Xia Chang

**Affiliations:** 1 Department of Neurology, Xishan People’s Hospital of Wuxi City, Wuxi, Jiangsu, China; 2 Department of Neurology, The Affiliated Zhangjiagang Hospital of Soochow University, Suzhou, Jiangsu, China

**Keywords:** thrombectomy, stroke, endothelial, dysfunction, prognosis

## Abstract

**Background and purpose:**

The Endothelial Activation and Stress Index (EASIX) is a marker of endothelial dysfunction. This study aimed to explore the association between the EASIX score and unfavorable outcome in patients with acute ischemic stroke (AIS) undergoing mechanical thrombectomy (MT) treatment.

**Methods:**

We enrolled AIS patients treated with MT from a multicenter study between June 2021 to September 2024. The EASIX score was calculated as lactate dehydrogenase (LDH) (U / L) × Creatinine (mg / dL) / Platelet Count (10 ^∧^ 9 / L). The unfavorable outcome was defined as 90-day modified Rankin scale score 3–6. We used multivariable logistic regression models to investigate the association between EASIX score and unfavorable outcome. A restricted cubic spline analysis was conducted to describe the linear relationship between the EASIX score and unfavorable outcome.

**Results:**

A total of 406 patients (258 [63.5%] male and median age: 71 years) were included in this study. 181 (44.6%) patients experienced unfavorable outcome. The logistic regression models demonstrated a robust association between the EASIX score and unfavorable outcome (log2 transformed odds ratio [OR]: 1.41; 95% confidence interval [CI]: 1.19–1.70; *P* < 0.001; tertile 3 vs. tertile 1 OR: 2.05; 95% CI: 1.14–3.55; *P* = 0.017). The restricted cubic spline analysis revealed a linearly increasing association between the EASIX score and the risk of unfavorable outcome (*P* for nonlinearity = 0.802).

**Conclusion:**

Higher EASIX levels were significantly related to unfavorable outcome in AIS patients treated with MT. EASIX score may serve as a valuable tool for early risk assessment and outcome prediction in AIS patients.

## Introduction

Mechanical thrombectomy (MT) is regarded as the standard of care for acute ischemic stroke (AIS) patients with large vessel occlusions (LVO) in the anterior circulation (Powers et al., 2019). MT can effectively recanalize occluded vessels in the early stages of AIS, thereby preserving the ischemic penumbra and reducing brain damage (Albers et al., 2018; Nogueira et al., 2018; Lapergue et al., 2021; Sarraj et al., 2024). However, MT is an invasive endovascular procedure that carries risks of complications such as hemorrhagic transformation and endothelial dysfunction, which may worsen ischemia-reperfusion injury (de la Riva et al., 2024). As a result, more than half of patients still fail to achieve functional independence at 90 days post-procedure (Goyal et al., 2016). The vascular endothelium plays a crucial role in maintaining normal brain function. It manages blood flow self-regulation, maintains the stability of the blood-brain barrier, and regulates immune-inflammatory responses (Madden, 2012). Previous studies reported that the increased endothelial dysfunction burden calculated from a standardized score of different biomarkers was associated with the risk of hemorrhagic complications after MT (Zhang et al., 2023). Plasma factor VII activating protease levels were significantly elevated in AIS patients with poor collateral circulation and were associated with worse neurological outcomes (Tian et al., 2022). However, endothelial dysfunction markers are not routinely tested in clinical practice due to their high cost. Therefore, there is a need for simpler, more accessible blood markers to assess endothelial dysfunction early after MT, helping clinicians identify high-risk patients and guide protective interventions.

The Endothelial Activation and Stress Index (EASIX) is a marker of endothelial dysfunction, calculated using the formula: lactate dehydrogenase (LDH) (U / L) × Creatinine (mg / dL) / Platelet Count (10 ^∧^ 9 / L). The EASIX score, first proposed by research teams in Germany and the United States, has proven to be a valuable tool for predicting prognosis in acute graft-versus-host disease following allogeneic hematopoietic stem cell transplantation. Studies demonstrated that EASIX is a strong predictor of overall survival (hazard ratio for a two-fold change: 1.23, *P* < 0.0001) and non-relapse mortality (cause-specific hazard ratio for a two-fold change: 1.24, *P* < 0.0001) (Luft et al., 2017). In critically ill patients with sepsis, the EASIX score has also been associated with an increased risk of 28-day mortality (Xu et al., 2023). The National Health and Nutrition Examination Survey indicated that EASIX may also act as a predictor for stroke prevalence and mortality outcome after stroke (Huang et al., 2025). However, limited research has examined the correlation between the EASIX score and unfavorable outcome after MT.

Hence, we conducted this multicenter study to explore the association between the EASIX score and unfavorable outcome in patients with AIS undergoing MT treatment.

## Materials and methods

Data that support the findings of this study were available from the corresponding authors upon reasonable request.

### Study population

This study is a retrospective analysis, involving patients with acute ischemic stroke enrolled from June 2021 to September 2024 at the Xishan People’s Hospital of Wuxi City and The Affiliated Zhangjiagang Hospital of Soochow University. Inclusion criteria for the study were: age 18 years or older; receiving MT within 24 h of symptom onset; imaging evidence of anterior circulation vessel occlusion (middle cerebral artery, anterior cerebral artery, internal carotid artery); pre-event modified Rankin Scale (mRS) score <2; Alberta Stroke Program Early Computed Tomography Score (ASPECTS) score ≥3; and National Institute of Health Stroke Scale (NIHSS) score ≥6. Exclusion criteria included: lack of laboratory test results at diagnosis such as serum LDH, serum creatinine, or platelet count; absence of follow-up information; and hematological tumor or autoimmune diseases that could affect blood test results.

The study received approval from the Institutional Review Board of Xishan People’s Hospital of Wuxi City (2025-0117) and was conducted in compliance with the Declaration of Helsinki. Informed consent was waived due to the retrospective nature of the study.

### Study design

Patient characteristics included age, gender, body mass index, smoking and drinking history, medical history, stroke type (assessed by the Trial of ORG 10172 in Acute Stroke Treatment) (Adams et al., 1993), stroke severity (assessed by the NIHSS score) (Brott et al., 1989), occlusion site, imaging parameters, intravenous thrombolysis and MT-related indicators, and hematological test results. Routine cranial imaging follow-up was conducted within 24–48 h postoperatively. The results were assessed by two experienced clinicians. Cerebral tissue ischemic status was evaluated using the ASPECTS scale (Barber et al., 2000). Successful recanalization was defined as the modified Thrombolysis in Cerebral Ischemia 2b/3 criteria. Patients routinely received antiplatelet therapy after the procedure, unless a high bleeding risk was identified based on clinical assessment. Blood samples were all measured upon admission. EASIX score calculated using the formula: LDH (U / L) × Creatinine (mg / dl] / platelet count (10 ^∧^ 9 / L) (Luft et al., 2017). Neutrophil to lymphocyte ratio (NLR) = neutrophil / lymphocyte count, platelet to lymphocyte ratio (PLR) = platelet / lymphocyte count.

### Functional outcome

Patients’ functional outcome was assessed using the mRS score. At 90 days after MT, clinicians evaluated patients via telephone or outpatient follow-up to inquire about their self-care abilities. Unfavorable outcome was defined as an mRS score of 3–6.

### Statistical analyses

Categorical variables were presented as frequencies and percentages, while continuous variables were described using median and interquartile range (IQR) or mean and standard deviation (SD). Normality of continuous variables was assessed using the Shapiro–Wilk test. Group differences were analyzed using *t*-test for normally distributed variables, Mann–Whitney test for non-normally distributed variables, and χ2 test or Fisher’s exact test for categorical variables. As the EASIX score distribution was skewed, we applied a log2 transformation. We then categorized the EASIX score using the tertiles (3.24 and 4.14) for analysis.

To elucidate the association between the EASIX score and unfavorable outcome after MT, we employed multiple logistic regression models for adjustment. Model 1 was the unadjusted model. Model 2 was adjusted for age, sex, smoke, and medical history including: hypertension, hyperlipidemia, diabetes mellitus, atrial fibrillation and coronary artery disease. Model 3 was adjusted for variables with *P* < 0.1 in the univariable analyses after the back-ward selection method with age, diabetes mellitus, hyperlipidemia, from puncture to reperfusion, baseline NIHSS, ASPECTS, mRS score, intravenous thrombolysis, number of attempts successful recanalization, and antiplatelet drug.

The predictive ability of the EASIX score for unfavorable outcome was evaluated using the receiver operating characteristic (ROC) curve and the area under the curve (AUC). Comparisons with NLR and PLR were made using the DeLong test. A restricted cubic spline analysis with 3 knots was conducted at the 10th, 50th, and 90th percentiles to describe the linear relationship between the EASIX score and unfavorable outcome. The net reclassification improvement (NRI) and integrated discrimination improvement (IDI) were used to assess the EASIX score’s ability to improve the model. Subgroup analyses were performed in the sensitivity analysis, including age, whether the stroke was wake-up stroke, TOAST classification, NIHSS score, and whether intravenous thrombolysis was administered.

All statistical tests were two-tailed, with a significance level of *P* = 0.05. All statistical analyses were performed using R version 4.2.2 (The R Foundation for Statistical Computing, Vienna, Austria).

## Results

### Clinical characteristics of the study population

As shown in Supplementary Figure 1, a total of 406 patients (258 [63.5%] male and median age: 71 IQR [64–79] years) were included in this study after excluding 246 patients due to lack of information and diseases. Among the study participants, 181 (44.6%) experienced unfavorable outcome, 151 (37.2%) underwent intravenous thrombolysis, 104 (25.6%) presented with wake-up strokes, and 186 (45.8%) had large artery atherosclerosis strokes. The mean duration from symptom onset to the procedure was 394.1 (± 286.7) minutes. Successful recanalization was achieved in 385 patients (94.8%), and 393 patients (96.8%) received antiplatelet therapy. Patients with unfavorable outcome exhibited a higher median age, a greater proportion of females, lower body mass, fewer smoking histories, a more prevalent history of diabetes and atrial fibrillation, a higher incidence of cardioembolic strokes, elevated NIHSS scores, reduced ASPECTS scores, extended intervals from puncture to successful recanalization, increased numbers of thrombectomy procedures, lower rates of successful recanalization (97.8% versus 91.2%), higher rates of antiplatelet therapy (98.7% versus 94.5%), and marked elevations in LDH and Creatinine levels, along with higher EASIX scores and increased inflammatory biomarkers, when contrasted with those who experienced favorable outcome (all *P* < 0.05; Table 1).

**TABLE 1:** Baseline characteristics of the study population.

Variable	Overall (*n* = 406)	Unfavorable outcome (*n* = 181)	Favorable outcome (*n* = 225)	*P*-value
Age (years)	71.0 [64.0, 79.0]	73.0 [67.0, 81.0]	69.0 [60.0, 78.0]	<0.001
Male, *n* (%)	258 (63.5)	105 (58.0)	153 (68.0)	0.048
Body mass index, (kg/m^2^)	23.9 [22.0, 26.7]	23.4 [21.3, 26.1]	24.2 [22.3, 27.0]	0.047
Smoking, *n* (%)	158 (38.9)	59 (32.6)	99 (44.0)	0.025
Drinking, *n* (%)	91 (22.4)	32 (17.7)	59 (26.2)	0.053
**Medical history, *n* (%)**				
Hypertension	287 (70.7)	133 (73.5)	154 (68.4)	0.318
Diabetes mellitus	112 (27.6)	63 (34.8)	49 (21.8)	0.005
Hyperlipidemia	40 (9.9)	12 (6.6)	28 (12.4)	0.074
Coronary artery disease	60 (14.8)	30 (16.6)	30 (13.3)	0.439
Atrial fibrillation	109 (26.8)	63 (34.8)	46 (20.4)	0.002
TOAST, *n* (%)				0.012
LAA	186 (45.8)	77 (42.5)	109 (48.4)	
CES	183 (45.1)	94 (51.9)	89 (39.6)	
Other	37 (9.1)	10 (5.5)	27 (12.0)	
**Baseline scores (points)**				
NIHSS	13 [10, 17]	14 [12, 18]	12 [9, 16]	<0.001
mRS	0 [0, 0]	0 [0, 0]	0 [0, 0]	<0.001
ASPECTS	9 [8, 9]	8 [8, 9]	9 [8, 9]	<0.001
Wake-up stroke, *n* (%)	104 (25.6)	52 (28.7)	52 (23.1)	0.240
IVT, *n* (%)	151 (37.2)	58 (32.0)	93 (41.3)	0.069
**Thrombectomy indictors**				
OTP (min)	394.1 (286.7)	404.8 (276.2)	385.5 (295.2)	0.497
PTR (min)	71.7 (40.6)	79.5 (43.5)	65.4 (37.1)	0.001
ASITN/SIR 2–3, *n* (%)	57 (14.0)	25 (13.8)	32 (14.2)	1.000
Number of attempts (n)	1 [1, 2]	2 [1, 3]	1 [1, 2]	0.001
Intra-arterial thrombolysis, *n* (%)	30 (7.4)	18 (9.9)	12 (5.3)	0.115
mTICI 2b / 3, *n* (%)	385 (94.8)	165 (91.2)	220 (97.8)	0.006
Occlusion site, *n* (%)				0.102
M1	205 (50.5)	79 (43.6)	126 (56.0)	
M2	34 (8.4)	18 (9.9)	16 (7.1)	
ICA	94 (23.2)	47 (26.0)	47 (20.9)	
T occlusion	73 (18.0)	37 (20.4)	36 (16.0)	
Antiplatelet therapy, *n* (%)	393 (96.8)	171 (94.5)	222 (98.7)	0.036
**Laboratory results**				
GLU (mmol/L)	7.9 (3.7)	7.9 (4.0)	8.0 (3.5)	0.833
BUN (mg/dL)	0.1 [0.1, 0.1]	0.1 [0.1, 0.1]	0.1 [0.1, 0.1]	0.003
Creatinine (mg/dL)	8.2 [6.8, 10.2]	8.7 [6.9, 11.0]	8.0 [6.7, 9.6]	0.008
Lactate dehydrogenase (U/L)	530.3 (1455.6)	774.5 (2130.9)	333.9 (308.1)	0.006
CRP (mg/L)	10.0 (3.9)	10.0 (4.0)	9.9 (3.9)	0.921
White blood cell count (× 10^9^/L)	8.2 (3.8)	8.8 (4.0)	7.7 (3.6)	0.007
Neutrophil count (× 10^9^/L)	1.2 (0.7)	1.2 (0.7)	1.2 (0.7)	0.274
Lymphocyte count (× 10^9^/L)	23.7 (34.9)	24.1 (37.1)	23.4 (33.1)	0.840
CRP (mg/L)	181.4 (67.7)	174.5 (63.6)	186.9 (70.5)	0.064
PLT count (× 10^9^/L)	7.9 (3.7)	7.9 (4.0)	8.0 (3.5)	0.833
NLR	7.2 [4.1, 12.6]	7.8 [4.7, 14.1]	6.4 [3.6, 12.2]	0.019
PLR	156.9 [106.5, 237.9]	164.9 [108.7, 257.6]	151.7 [105.7, 230.0]	0.231
EASIX	12.7 [8.2, 23.3]	15.2 [9.2, 29.2]	11.5 [7.3, 19.5]	<0.001

Values are presented as median [interquartile range] for continuous variables with non-normal distributions, and as mean (standard deviation) for continuous variables with normal distributions. ASITN / SIR, the American Society of Interventional and Therapeutic Neuroradiology/Society of Interventional Radiology; ASPECTS, the Alberta Stroke Program Early Computed Tomography Score; CES, cardioembolism; CRP, C-reactive protein; EASIX, endothelial activation and stress index; GLU, fasting blood glucose; ICA, internal carotid artery; IVT, intravenous thrombolysis; LAA, large artery atherosclerosis; mRS, modified Rankin Scale; MT, mechanical thrombectomy; mTICI, modified Thrombolysis in Cerebral Infarction Score; NIHSS, National Institute of Health Stroke Scale; NLR, neutrophil / lymphocyte; OTP, from onset to puncture; PLR, platelet / lymphocyte; PTR, from puncture to recanalization; TOAST, the trial of ORG 10172 in Acute Stroke Treatment classification.

### Logistical regression models

**FIGURE 1:**
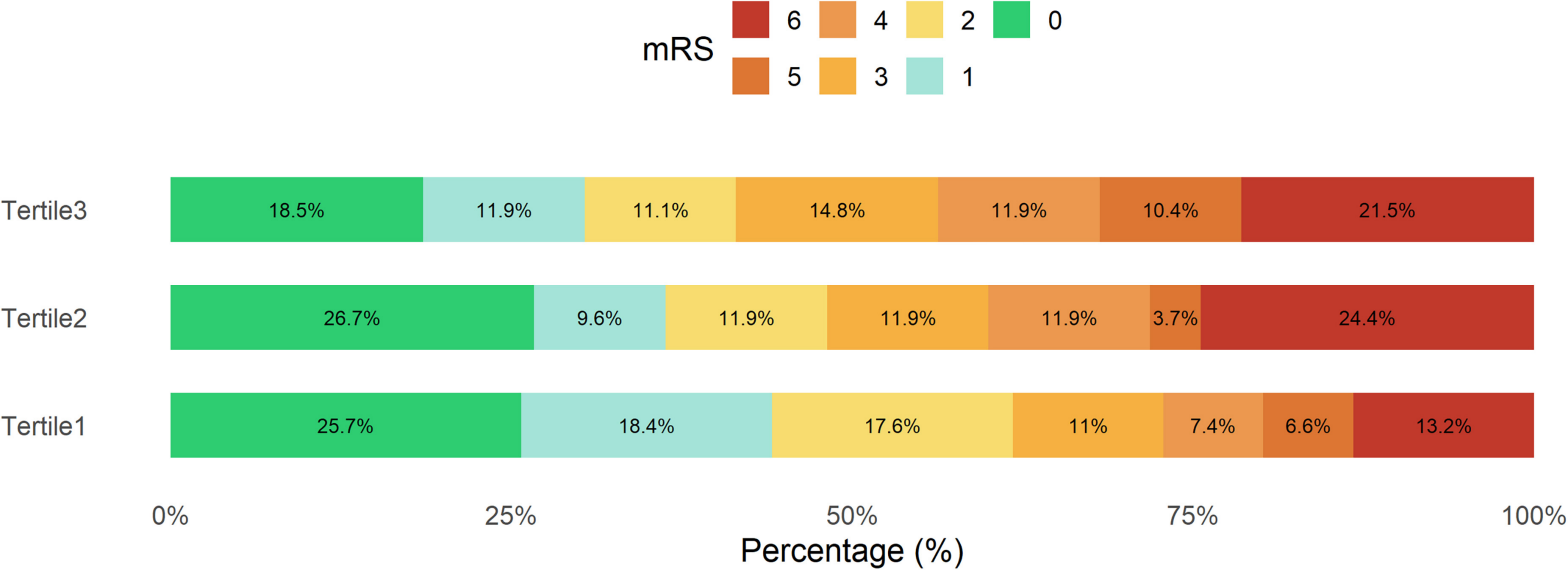
The distribution of the functional outcome after MT categorized by tertiles of EASIX. EASIX, endothelial activation and stress index; mRS, modified Rankin scale; MT, mechanical thrombectomy. Patients with tertile 3 EASIX had a higher proportion of mRS 3–6 than tertile 2 and tertile 1.

As shown in Figure 1, patients with tertile 3 EASIX had a higher proportion of mRS 3–6 than tertile 2 and tertile 1. The logistic regression models consistently demonstrated a robust association between the EASIX score and unfavorable outcome after MT. In model 1, the EASIX score was significantly associated with unfavorable outcome (log2 transformed odds ratio [OR]: 1.42; 95% confidence interval [CI]: 1.22–1.67; *P* < 0.001; tertile 3 vs. tertile 1 OR: 2.16; 95% CI: 1.33–3.55; *P* = 0.002). This finding was echoed in model 2 (log2 transformed OR: 1.42; 95% CI: 1.21–1.68; *P* < 0.001; tertile 3 vs. tertile 1 OR: 2.05; 95% CI: 1.23–3.44; *P* = 0.006) and model 3 (log2 transformed OR: 1.41; 95% CI: 1.19–1.70; *P* < 0.001; tertile 3 vs. tertile 1 OR: 2.02; 95% CI: 1.15–3.59; *P* = 0.015), further reinforcing the predictive significance of the EASIX score in this context (Table 2).

**TABLE 2:** Logistic regression analyses for the association between EASIX score and unfavorable outcome after MT.

EASIX	Model 1		Model 2		Model 3	
	OR (95% CI)	*P* value	OR (95% CI)	*P*-value	OR (95% CI)	*P*-value
Log2 transformed	1.42 (1.22–1.67)	<0.001	1.42 (1.21–1.68)	<0.001	1.41 (1.19–1.70)	0.001
Tertile 1	Reference		Reference		Reference	
Tertile 2	1.61 (0.99–2.63)	0.057	1.45 (0.87–2.44)	0.159	1.60 (0.91–2.83)	0.106
Tertile 3	2.16 (1.33–3.55)	0.002	2.05 (1.23–3.44)	0.006	2.02 (1.15–3.59)	0.015

ASPECTS, the Alberta Stroke Program Early Computed Tomography Score; CI, confidence interval; EASIX, endothelial activation and stress index; IVT, intravenous thrombolysis; mRS, modified Rankin scale; MT, mechanical thrombectomy; NIHSS, National Institute of Health Stroke Scale; OR, odds ratio; PTR, from puncture to recanalization. Model 1 was the unadjusted model. Model 2 was adjusted for age, sex, smoke, and medical history including: hypertension, diabetes mellitus, hyperlipidemia, coronary artery disease and atrial fibrillation. Model 3 was adjusted for variables with *P* < 0.1 in the univariable analyses after the back-ward selection method with age, diabetes mellitus, hyperlipidemia, PTR, baseline NIHSS, ASPECTS, mRS, IVT, number of attempts, successful recanalization, and antiplatelet drug.

### EASIX score and unfavorable outcome

**FIGURE 2:**
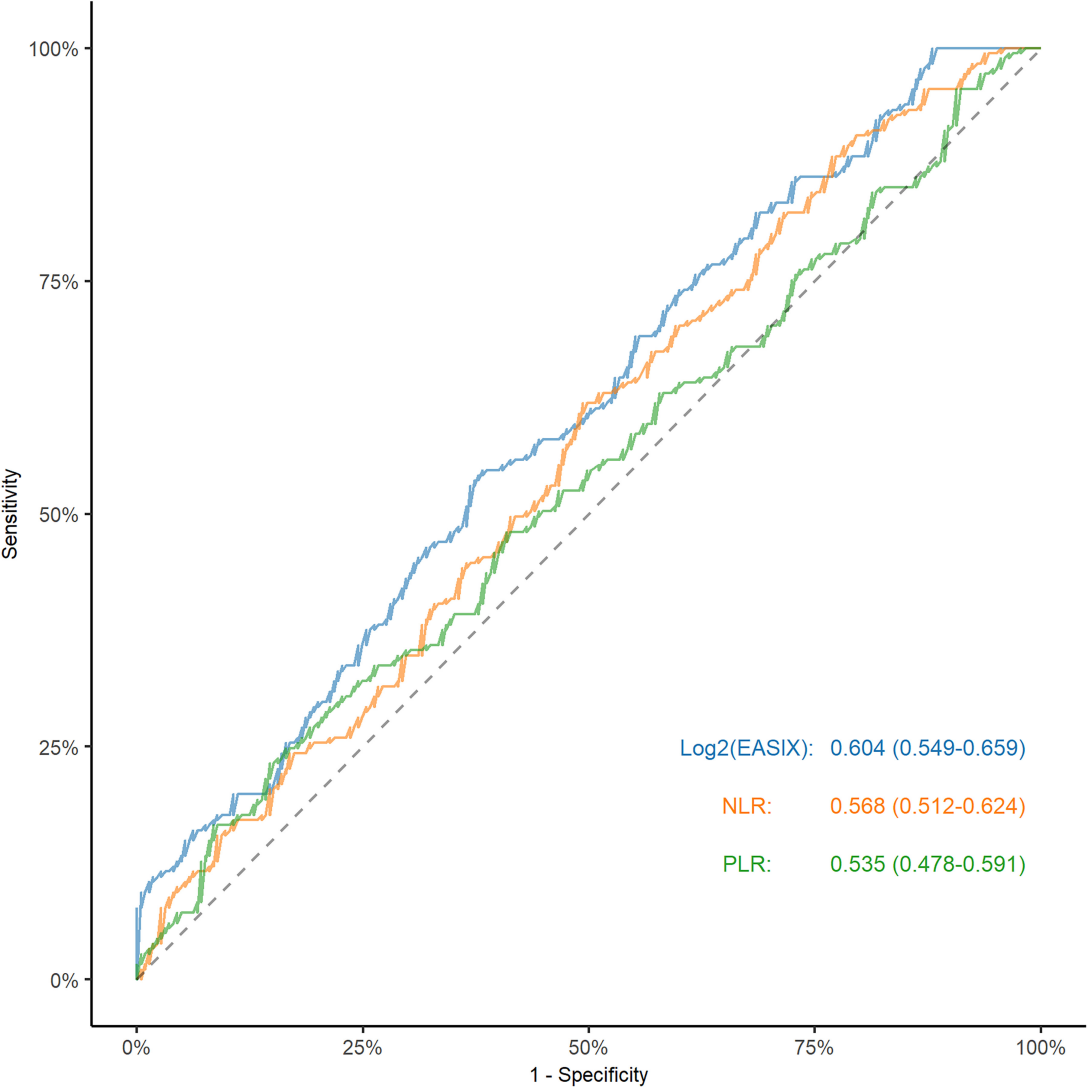
Comparative analysis of the discrimination of EASIX and inflammation-related markers for unfavorable outcome after MT. EASIX, endothelial activation and stress index; MT, mechanical thrombectomy; NLR, neutrophil / lymphocyte; PLR, platelet / lymphocyte. The curves represented the receiver operating characteristic curves for EASIX and inflammation-related markers. EASIX had the highest area under curve values (*P* value for Delong test with NLR = 0.330 and PLR = 0.094).

**FIGURE 3:**
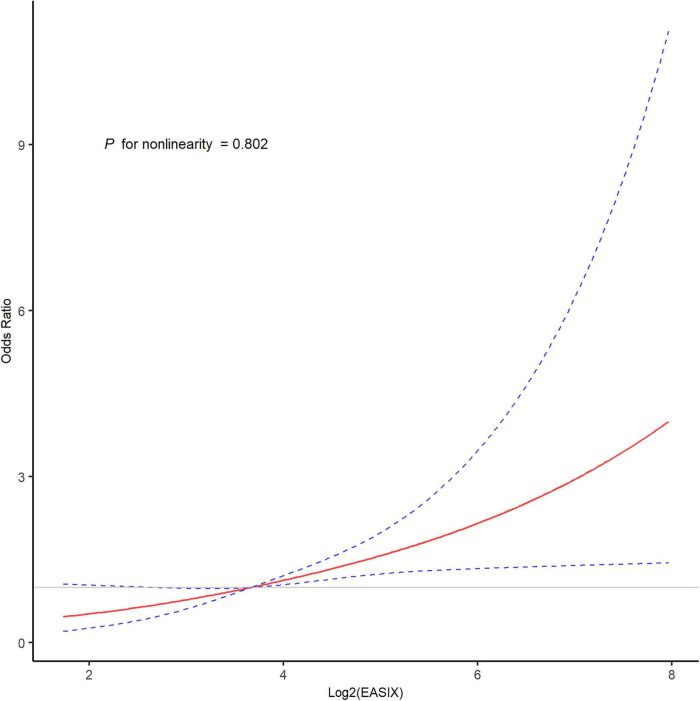
The restricted cubic spline curve for the association of EASIX with unfavorable outcome after MT. EASIX, endothelial activation and stress index; MT, mechanical thrombectomy. The restricted cubic spline showed a linear relationship between EASIX and the risk of unfavorable outcome. The red line represented the odds ratio and dashed lines represented the 95% confidence intervals.

The ROC curve indicated that the EASIX score exhibited the highest AUC (0.604, 95% CI 0.549–0.659; Delong test with NLR = 0.330 and PLR = 0.094; Figure 2). The restricted cubic spline analysis revealed a linearly increasing association between EASIX and the risk of unfavorable outcome (*P* for nonlinearity = 0.802; Figure 3). Incorporating EASIX into model 2 enhanced the model’s predictive power (NRI: 0.187; 95% CI: 0.066–0.393; *P* = 0.032; IDI: 0.046; 95% CI, 0.025–0.068; *P* < 0.001), and similarly, in model 3 (NRI: 0.150; 95% CI: 0.054–0.344; *P* = 0.041; IDI: 0.036; 95% CI, 0.017–0.054); *P* < 0.001; Supplementary Table 1). Subgroup analyses did not reveal any significant interaction effects for age, wake-up stroke, TOAST classification, NIHSS score, and intravenous thrombolysis (all *P* > 0.05; Supplementary Figure 2).

## Discussion

To the best of our knowledge, this is the first study to investigate the association between EASIX score and unfavorable outcome in AIS patients undergoing MT. We found that higher EASIX scores were independently associated with an increased risk of the unfavorable 90-day outcome, with consistent results across multiple subgroups.

Endothelial dysfunction played an important role in patients with ischemic stroke. An observational study involving 637 participants utilized reactive hyperemia peripheral arterial tonometry to assess markers of microvascular endothelial function. The study revealed a significantly higher incidence of stroke in patients with endothelial dysfunction compared to those without (log-rank *P* = 0.03) during follow-up (Toya et al., 2020). Tuttolomondo et al. found that endothelial function was significantly associated with increased arterial stiffness in AIS patients. Furthermore, the degree of endothelial injury in patients with large artery atherosclerosis was higher, as indicated by the reactive hyperemia index, thereby aiding in the classification of stroke subtypes (Tuttolomondo et al., 2017). Bima et al. conducted a case-control study measuring asymmetric dimethylarginine and symmetric dimethylarginine, both recognized as mediators of endothelial dysfunction. They found that high-risk patients exhibited significantly reduced levels of endothelial function markers, which were associated with adverse clinical outcomes, including prolonged hospitalization and delayed recovery (Bima et al., 2024). However, current tests for endothelial function are complex and costly, limiting their applicability in routine clinical practice. Thus, there is a need for simpler and more reliable markers to assess endothelial function in AIS patients.

EASIX was initially developed and validated as a reliable predictor of overall survival in patients with steroid-refractory graft-versus-host disease following allogeneic stem cell transplantation — a condition often linked to thrombotic microangiopathy from endothelial dysfunction (Luft et al., 2017). Since then, its prognostic value, based on LDH, serum creatinine, and platelet count, has been validated across diverse patient populations, including those with multiple myeloma (Song et al., 2020), diffuse large B-cell lymphoma (Park et al., 2022), small cell lung cancer (Go et al., 2022), acute myocardial infarction (Sang et al., 2024), and heart failure (Yin and Wang, 2025). Moreover, Huang et al. found that the EASIX score was positively associated with stroke risk after full adjustment, and demonstrated a non-linear relationship between EASIX levels and both stroke occurrence and mortality outcomes (Huang et al., 2025). Our study demonstrated a significant association between EASIX score and unfavorable outcome at 90 days post-MT surgery. However, the predictive performance of EASIX alone was limited, showing only poor to fair discriminative ability as an independent predictor. Therefore, EASIX may be more appropriately used as a component of a comprehensive predictive model rather than as a standalone prognostic tool.

Potential mechanisms explaining the association between EASIX and unfavorable outcome in AIS patients after MT treatment may include: Firstly, our study reveals that the EASIX score exhibits superior predictive power compared to inflammatory markers. Previous research has elucidated that endothelial cell-associated molecules, such as cell adhesion molecules, are central to vascular dysfunction and tissue injury, with their activities deeply intertwined with signaling pathways including MAP-kinase p38, Akt/PKB, NF-κB, and ERK1/2, which are integral to inflammatory processes (Tuttolomondo et al., 2020). Secondly, after vascular endothelial injury, various molecules—including LDH, platelets, and lymphocytes—are involved in the repair process. Elevated LDH levels, in particular, reflect the severity of endothelial damage and are associated with blood-brain barrier disruption and other factors influencing AIS prognosis (Chopra et al., 1987). Renal impairment is associated with an elevated risk of functional independence post-MT, and serum creatinine levels serve as an indicator of renal function (Xiao et al., 2020). Thirdly, vascular remodeling and microcirculatory deficits, stemming from endothelial dysfunction, lead to reduced vascular compliance and compromised autoregulation. Consequently, this exacerbates infarct size, lethality, and impairs functional recovery after the stroke (Tregub et al., 2022).

However, our study has certain limitations. Firstly, as a multicenter study, there may be variations in treatment methods and hematological detection indicators. Secondly, being a retrospective study, we were unable to obtain the dynamic changes of the EASIX score, which may be more persuasive. Thirdly, while other hematological endothelial markers were not collected due to the trial design, we used predictive inflammatory markers for comparison, and EASIX still demonstrated accuracy. Forth, although we attempted to minimize potential biases through multivariable adjustments, residual confounding may still exist. Factors such as comorbidities (e.g., heart failure, baseline renal dysfunction), stroke etiology, and detailed imaging assessments of the infarct core and penumbra could affect both EASIX levels and functional outcomes. Finally, as the study predominantly included patients from southern China, the findings may not be applicable for generalization to other ethnic populations.

## Conclusion

Higher EASIX levels were significantly related to unfavorable outcome in AIS patients treated with MT for LVO. The EASIX score may serve as a valuable tool for early risk assessment and outcome prediction in AIS patients with anterior circulation occlusion and ASPECTS ≥3; however, its applicability to patients with posterior circulation strokes or large infarcts may be limited.

## Data Availability

The original contributions presented in this study are included in this article/Supplementary material, further inquiries can be directed to the corresponding author.
