# Supplementary material for: Prognostic value of endothelial activation and stress index in mechanical thrombectomy for patients with acute ischemic stroke
Source: Front Aging Neurosci. 2025 Dec 10;17:1683690. doi: 10.3389/fnagi.2025.1683690 (PMC12727912; doi:10.3389/fnagi.2025.1683690)
Supplement: Supplementary file 1 [file Data_Sheet_1.pdf]

Supplementary materials

Figure 1. Flow chart of the study.

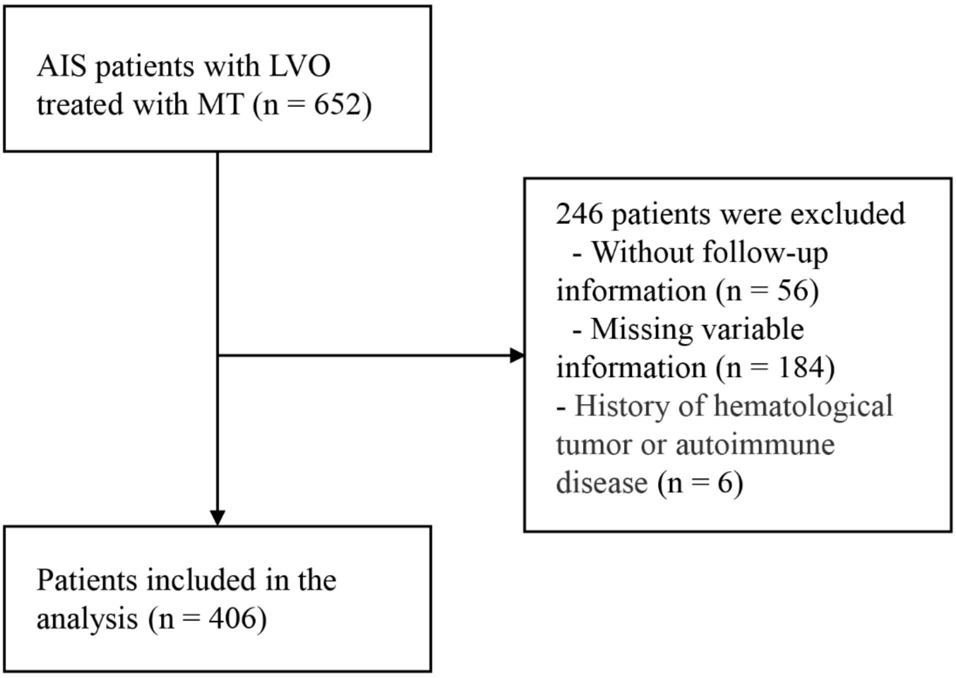

Abbreviations: AIS, acute ischemic stroke; LVO, large vessel occlusion; MT, mechanical thrombectomy.

Figure 2. Subgroup analyses for EASIX.

Subgroup	No of patients	OR (95% CI)	*P* for interaction
**Age**			**0.794**
≤70	199	1.436 (1.156-1.842)	
>70	207	1.377 (1.119-1.724)	
**Wake up**			**0.964**
No	302	1.415 (1.186-1.711)	
Yes	104	1.427 (1.076-2.006)	
**TOAST**			**0.844**
CES	183	1.351 (1.081-1.725)	
LAA	186	1.506 (1.202-1.950)	
Other	37	1.118 (0.620-2.055)	
**NIHSS**			**0.394**
≤15	269	1.322 (1.097-1.622)	
>15	137	1.530 (1.184-2.053)	
**IVT**			**0.726**
No	255	1.456 (1.210-1.790)	
Yes	151	1.370 (1.040-1.831)	

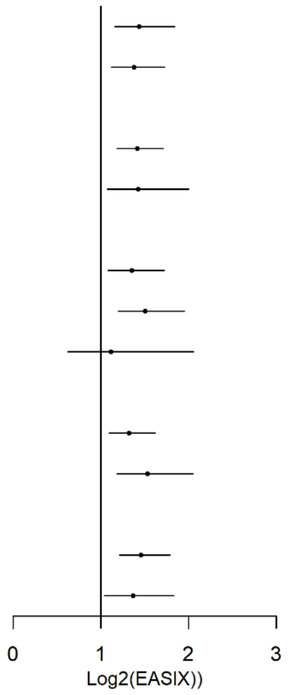

Abbreviations: CES, cardioembolism; CI, confidence interval; EASIX, endothelial activation and stress index; IVT, intravenous thrombolysis; LAA, large artery atherosclerosis; NIHSS, National Institute of Health Stroke Scale; OR, odds ratio; TOAST, the trial of ORG 10172 in Acute Stroke Treatment classification.

Table 1. Reclassification indexes for EASIX and unfavorable outcome after MT.

Models	NRI (95% CI)	*P* value	IDI (95% CI)	*P* value
Model 2	0.187 (0.066-0.393)	0.032	0.046 (0.025-0.068)	<0.001
Model 3	0.150 (0.054-0.344)	0.041	0.036 (0.017-0.054)	<0.001

Abbreviations: ASPECTS, the Alberta Stroke Program Early Computed Tomography Score; CI, confidence interval; EASIX, endothelial activation and stress index; IDI, integrated discrimination improvement; IVT, intravenous thrombolysis; mRS, modified Rankin scale; MT, mechanical thrombectomy; NIHSS, National Institute of Health Stroke Scale; NRI, net reclassification index; OR, odds ratio; PTR, from puncture to recanalization. Model 2 was adjusted for age, sex, smoke, and medical history including: hypertension, diabetes mellitus, hyperlipidemia, coronary artery disease and atrial fibrillation. Model 3 was adjusted for age, diabetes mellitus, hyperlipidemia, PTR, NIHSS, ASPECTS, mRS, IVT, number of attempts, successful recanalization and antiplatelet drug.
